# The effect of minocycline on the masticatory movements following the inferior alveolar nerve transection in freely moving rats

**DOI:** 10.1186/1744-8069-8-27

**Published:** 2012-04-20

**Authors:** Rahman Md Mostafeezur, Hossain Md Zakir, Yoshiaki Yamada, Kensuke Yamamura, Koichi Iwata, Barry J Sessle, Junichi Kitagawa

**Affiliations:** 1 Division of Oral Physiology, Department of Oral Biological Science, Niigata University Graduate School of Medical and Dental Sciences, 2-5274, Gakkocho-dori, Niigata, 951-8514, Japan; 2 Department of Physiology, Nihon University School of Dentistry, 1-8-13 Kandasurugadai, Chiyoda-ku, Tokyo, 101-8310, Japan; 3 Faculty of Dentistry, University of Toronto, 124 Edward Street, Toronto, ON, M5G 1G6, Canada

**Keywords:** Minocycline, microglia, trigeminal neuropathic pain, trigeminal motor nucleus, trigeminal principal sensory nucleus, mastication, modulation

## Abstract

**Background:**

To determine the effects of inferior alveolar nerve transection (IAN-X) on masticatory movements in freely moving rats and to test if microglial cells in the trigeminal principal sensory nucleus (prV) or motor nucleus (motV) may be involved in modulation of mastication, the effects of microglial cell inhibitor minocycline (MC) on masticatory jaw movements, microglia (Iba1) immunohistochemistry and the masticatory jaw movements and related masticatory muscle EMG activities were studied in IAN-X rats.

**Results:**

The number of Iba1-immunoreactive (IR) cells both in prV and motV was significantly larger in IAN-X rats compared with sham rats on day 3 after IAN-X. The intraperitoneal (i.p.) administration of MC caused a significant reduction of the number of Iba1-IR cells both in prV and motV that was evident on day 14 after IAN-X. Furthermore, a significant reduction of the number of Iba1-IR cells could be observed in motV but not in prV after microinjection (m.i.) of MC into the motV of IAN-X rats. The rats also exhibited a significant decrease in the head-withdrawal threshold on the side ipsilateral to the IAN-X compared to the threshold before IAN-X and it lasted to day 14. In addition, IAN-X markedly affected the ability to rat to carry out mastication. The number of complete masticatory sequences was significantly decreased. Furthermore, the total masticatory sequence time and food preparatory (PP) period duration was significantly elongated in compared to sham rats. Although IAN-X significantly affected the total number of chewing cycles within the RC period of a masticatory sequence, it had no effect on the duration of the chewing cycles. On the other hand, systemic administration of MC (both i.p. and m.i.) in IAN-X rats significantly improved decreased head-withdrawal threshold and the impaired masticatory jaw movements.

**Conclusions:**

The present findings reveal that the strong modulation of masticatory jaw movements occurs following microglial cell activation after IAN-X, and the modulation recovers after inhibition of the microglial cell activation by MC, suggesting that microglial cell activation in the motV as well as in the prV has a pivotal role in modulating mastication following trigeminal nerve injury associated with orofacial neuropathic pain.

## Background

It is well known that jaw movements during mastication activate various intraoral sensory receptors that are involved in providing sensory feedback to the brain to guide masticatory jaw movements [1-4], sensory inputs related specifically to pain can modulated masticatory movements and other jaw motor functions [5-11]. Thus, noxious inputs from intraoral tissues can modulate masticatory movements. However, little is known about the mechanisms by which chronic orofacial pain may affect masticatory movements. Orofacial persistent pain is frequently observed following trauma of the trigeminal nerve. It is however now clear that trigeminal nerve injury can produce a neuropathic pain condition that is associated with central sensitization of trigeminal brainstem nociceptive neurons on subnucleus caudalis (Vc) [12-15], and that the normal hyperexcitability depends on the functional insights of glia [14,16-19].

Indeed, in addition to the importance of neuronal mechanisms underlying orofacial neuropathic pain and modulation of motor functions, there is considerable evidence that glial cells in pain signaling pathways may play an important role both in sensory and motor functions. While hyperactive glial cells have been reported mainly in the Vc and upper cervical dorsal horn (C1-C2) after peripheral nerve injury or inflammation and have been recognized for their role in initiating or maintaining neuropathic pain, it has also been reported that glial cells are activated surrounding the prV [19] and facial motor nuclei [20] following peripheral nerve injury.

Furthermore, other recent evidence suggests that glial cells may play an important role in controlling the activity of the neuronal networks underlying motor functions [21-23]. However, no studies have been conducted in animal neuropathic pain models to evaluate whether trigeminal nerve injury affects masticatory movements and whether glial cells are involved in the modulation of orofacial motor functions after the nerve injury. We hypothesized that microglial cells in or adjacent to the motV as well as the prV are activated after IAN injury and such hyperactive microglial cells might be involved in the nerve injury-induced modulation of masticatory movements.

MC, tetracycline derivative, is known to attenuate the abnormal pain behavior by the inhibition of the microglial activity [24-33], and thereby improve the motor behaviors [28]. Our hypothesis was that administration of MC may also be effective for the improvement of masticatory movements after IAN-X.

Thus, the aim of the present study was (1) to determine the effects of IAN-X on masticatory movements in freely moving rats, (2) to test if microglia in the prV or motV may be involved in modulation of these effects and (3) the effect of MC on the masticatory movements following the IAN-X in freely moving rats.

## Results

### Microglial cell activation in prV and motV

**Figure 1:**
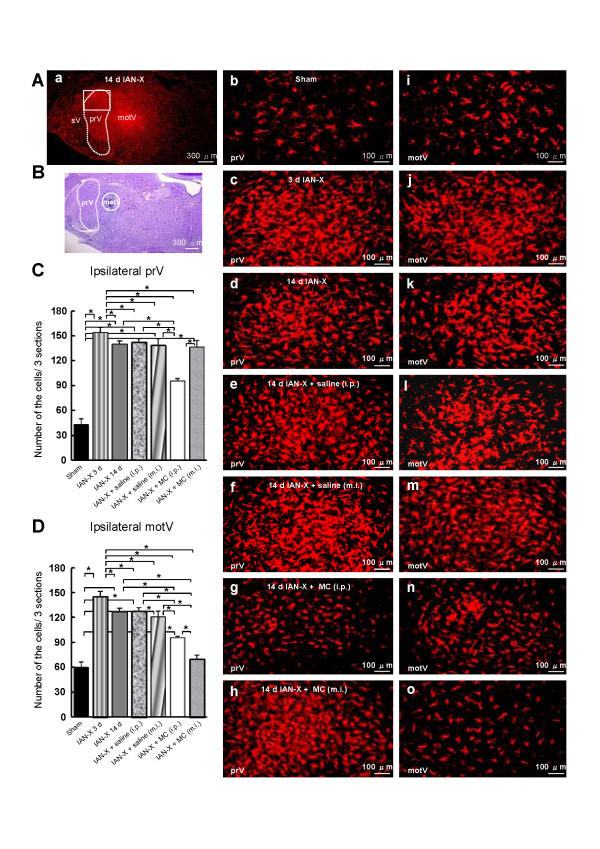
**A: Temporal profile of the microglial cell activation in the prV and motV.** In the case of IAN-X rats, microglial cells were immuno-labeled with Iba1 in the prV and motV at 3 (n = 5) and 14 day (n = 5) after IAN-X. In the sham (n = 5) or IAN-X + MC-injected (both the i.p., n = 5 and m.i., n = 5) or IAN-X + saline-injected rats (i.p., n = 5 and m.i., n = 5), microglial cells were immuno-labeled after 14 days. **B**: Localization of the m.i. site in the motV. The number of Iba1-immunoreactive (IR) cells in the three sections per rats (**C-D**). The Iba1-IR cells were increased at 3 day after IAN-X both in the prV (**C**) and motV (**D**). After 14 days, a small decrease in these cells was observed in IAN-X rats. The i.p. administration of MC strongly attenuated the number of Iba1-IR cells both in the prV and motV. It is notable that m.i. into motV strongly attenuated the number of Iba1-IR cell in the motV but not in the prV. (*) denotes the significant difference between experimental groups (*p* < 0.05, one way ANOVA with tukey test).

The Iba1 immunohistochemical study was conducted to visualize activated microglial cells, and the distribution pattern of Iba1-immunoreactive (IR) cells was studied in IAN-X rats (Figure 1). Some previous studied have reported that Iba1-IR cells are found in the Vc [17-19], but the present study focused on prV and motV. A large number of Iba1-IR cells were observed both in the prV and motV in IAN-X rats (Figure 1A, C, D). The number of Iba1-IR cells was significantly larger in IAN-X rats compared with sham rats on day 3 after IAN-X both in the prV and motV (Figure 1Ab, Ai, Ac, Aj, 1C-D) and was slightly reduced on day 14 (Figure 1Ad, Ak, 1C-D).

Since MC is known to attenuate the activation of microglial cells in the spinal dorsal horn after spinal cord injury [27,28,31,34]), we studied the effect of i.p. or m.i. of MC into the motV on Iba1-IR cell expression in IAN-X rats (Figure 1A, C, D). The i.p. administration of MC caused a significant reduction of the number of Iba1-IR cells both in the prV and motV on day 14 after IAN-X (Figure 1Ag, An, 1C-D). Furthermore, a significant reduction of the number of the Iba1-IR cells was observed in the motV after m.i. of MC into the motV (Figure 1Ao, 1D). On the other hand, we could not observe any significant changes in the number of Iba1-IR cells in the prV after m.i. of MC into the motV (Figure 1Ah, 1C), indicating that the injected MC was not extending into the prV. In addition, no significant changes in the number of the Ib1-IR cells was observed both in the prV and motV after saline administration (i.p. or m.i. into the motV) in IAN-X rats (Figure 1Ae, Al, Af, Am, 1C-D).

### Distribution of FG-labeled neurons in TG

FG tracing study of the trigeminal ganglion neurons was conducted for evidence suggesting the regeneration and reinnervation by the transected IAN [15,35]. FG-labeled neurons were localized in the root of the third branch of the trigeminal nerve (Additional File 1: Figure S1B, S1C). In sham rats, a large numbers of FG-labeled neurons were observed in the TG (Additional File 1: Figure S1Bb, S1C). No or few labeled neurons were observed at day 3 after IAN-X (Additional File 1: Figure S1Bc, S1C). However, the number of FG-labeled neurons was increased at day 7 after IAN-X in compared with that at day 3 after IAN-X (Additional File 1: Figure S1Bd, S1C). The large number of FG-labeled neurons was apparent in the TG at day 14 after IAN-X (Additional File 1: Figure S1Be, S1C). It is notable that we could not observe any FG-labeled neurons in the root of the second branch of the trigeminal nerve at day 14 after IAN-X (Additional File 1: Figure S1Ba).

### Head-withdrawal threshold to mechanical stimulation of mental skin

**Figure 2:**
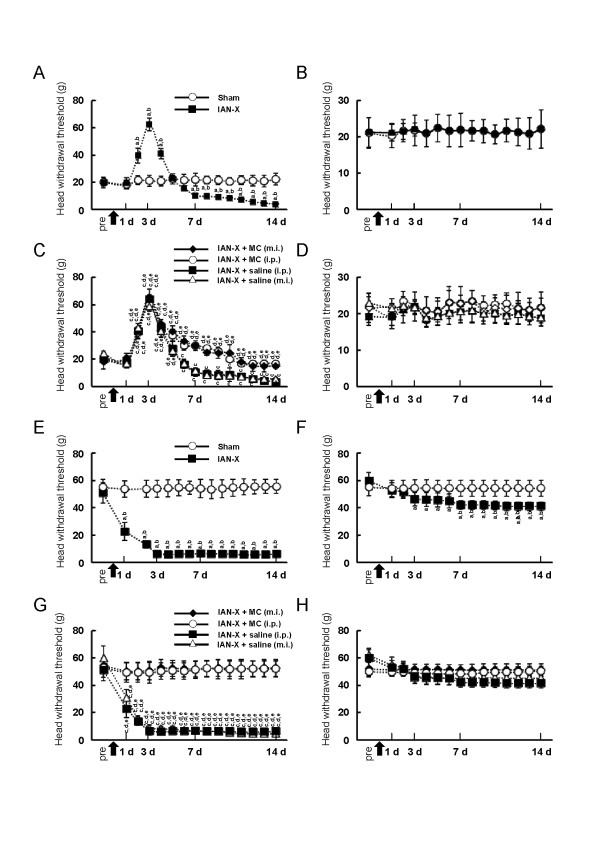
**Change in the head-withdrawal threshold to mechanical stimulation of the mental skin and whisker pad skin. A**: Mechanical head-withdrawal threshold in the mental skin ipsilateral to the sham operation (n = 12) and IAN-X (n = 12), **B**: Mechanical head-withdrawal threshold in the mental skin contralateral to the sham operation and IAN-X, **C**: Mechanical head-withdrawal threshold in the mental skin ipsilateral to IAN-X after MC (i.p., n = 12 and m.i., n = 10) and saline (i.p., n = 10 and m.i., n = 10) administration, **D**: Mechanical head-withdrawal threshold in the mental skin contralateral to IAN-X after MC (i.p., and m.i.,) and saline (i.p., and m.i.,) administration, **E**: Mechanical head-withdrawal threshold in the whisker pad skin ipsilateral to the sham operation (n = 12) and IAN-X (n = 12), **F**: Mechanical head-withdrawal threshold in the whisker pad skin contralateral to the sham operation and IAN-X, **G**: Mechanical head-withdrawal threshold in the whisker pad skin ipsilateral to IAN-X after MC (i.p., n = 12 and m.i., n = 10) and saline (i.p., n = 10 and m.i., n = 10) administration, **H**: Mechanical head-withdrawal threshold in the whisker pad skin contralateral to IAN-X after MC (i.p., and m.i.,) and saline (i.p., and m.i.,) administration. The arrow in the graph indicates the time point of the sham operation or IAN-X. The behavioral test was assessed once a day for 14 days, and the median value was adopted. It is notable that the mechanical head-withdrawal threshold was significantly decreased 14 days after IAN-X, and administration of MC (both i.p. and m.i.) significantly attenuated the reduction on the head-withdrawal threshold 14 days after IAN-X. Pre: the day before sham or IAN-X, a indicates: pre-IAN-X vs. post-IAN-X, b indicates: sham vs. IAN-X, c indicates: pre-IAN-X + MC (i.p.) vs. post-IAN-X + MC (i.p.), pre-IAN-X + MC (m.i.) vs. post-IAN-X + MC (m.i.), pre-IAN-X + saline (i.p.) vs. post-IAN-X + saline (i.p.), pre-IAN-X + saline (m.i.) vs. post-IAN-X + saline (m.i.), d indicates: IAN-X + MC (i.p.) vs. IAN-X + MC (m.i.), IAN-X + MC (i.p.) vs. IAN-X + saline (i.p.) and IAN-X + MC (i.p.) vs. IAN-X + saline (m.i.), e indicates: IAN-X + MC (m.i.) vs. IAN-X + saline (i.p.) and IAN-X + MC (m.i.) vs. IAN-X + saline (m.i.), p < 0.05 was considered significant (one way ANOVA with Tukey test, and Dunnett’s test for pre vs. post IAN-X or pre vs. post IAN-X + MC or pre vs. post IAN-X + saline).

During behavioral testing, rats stayed in the plastic cage quietly and protruded their nose through the hole in the front wall of the cage without any discomfort. Rats also did not show vocalization or autotomy during measurement of the head-withdrawal threshold to mechanical stimulation of the mental skin. Before the IAN-X operation (pre in Figure 2A, B), the mean mechanical head-withdrawal threshold was 20.0 +/− 4.0 g (mean +/− SD, n = 12) on the side ipsilateral to the IAN-X and 22.0 +/− 3.6 g on the side contralateral to the IAN-X. After the IAN-X, the mechanical head-withdrawal threshold on the side ipsilateral to the IAN-X was significantly increased (62.4 +/− 4.6 g, n = 12) on day 3 after the IAN-X compared to that before the IAN-X operation and compared to that in sham rats (before operation: 20 +/− 4.0 g, sham: 20.2 +/− 3.0, n = 12 in each) (Figure 2A), whereas no significant change in the head-withdrawal threshold was observed on the side contralateral to the IAN-X (22.2 +/− 3.4 g, n = 12) at day 3 after IAN-X (Figure 2B). Four days after the IAN-X, the head-withdrawal threshold began to decrease, and a significant decrement in the head-withdrawal threshold could be observed in the side ipsilateral to the IAN-X on day 7 compared with that before operation (before IAN-X: 20 +/− 4.0 g, IAN-X on day 7 :10.2 +/− 2.0 g, n = 10), and lasted until day 14 (Figure 2A). On the other hand, there was no significant change in the head-withdrawal threshold on the side contralateral to the IAN-X and in sham rats (Figure 2A, B).

To examine the effect of MC on nocifensive behavior following the IAN-X, we measured the head-withdrawal threshold to mechanical stimulation of the mental skin in IAN-X rats after i.p. injection of MC (n = 12) and after m.i. injection of MC into the motV (n = 10) (Figure 2C, D). The head-withdrawal threshold to mechanical stimulation of the mental skin in IAN-X + saline-injected rats (i.p., or m.i. into the motV) on day 14 after IAN-X was significantly lower than that before the IAN-X operation (saline (i.p.): 3.3 +/− 2.0 g, saline (m.i.): 3.9 +/− 1.7 g, before IAN-X: 20.1 +/− 3.5 g, n = 10) (Figure 2C). On the other hand, on day 14 after the daily administration of MC (i.p., or m.i. into the motV), the reduction of the head-withdrawal threshold to mechanical stimulation of the mental skin in IAN-X rats was significantly attenuated (MC (i.p.) : 17.0 +/− 2.6 g, MC (m.i.): 15.2 +/− 1.0 g) (Figure 2C). There was no significant influence of MC or saline on the head-withdrawal threshold on the side contralateral to the IAN-X or sham operation (Figure 2D).

### Head-withdrawal threshold to mechanical stimulation of whisker pad skin

Our previous study documented that mechanical hypersensitivity was developed in the whisker pad skin innervated by the infra-orbital nerve at the early period after IAN-X [13]. Thus, the mechanical head-withdrawal threshold was also investigated in the whisker pad skin. Before the IAN-X, the mean mechanical head-withdrawal threshold was 51.0 +/− 8.0 g (mean +/− SD, n = 12) on the side ipsilateral to the IAN-X and 59.0 +/− 6.0 g on the side contralateral to the IAN-X (Figure 2E2F). After the IAN-X, the head-withdrawal threshold to mechanical stimulation of whisker pad was significantly decreased on day 3 in the side ipsilateral to the IAN-X compared with sham rats (IAN-X: 5.1 +/− 2.4 g, sham: 54.0 +/− 6.1 g) (Figure 2E). The significant decrement of the head-withdrawal threshold was observed at least 14 days after IAN-X (IAN-X: 6.1 +/− 1.0 g) (Figure 2E). In addition, a weak reduction of head-withdrawal threshold was observed on the side contralateral to the IAN-X (Figure 2F).

After administration of MC (i.p. or m.i. into the motV) once daily for 14 days, the reduction of head-withdrawal threshold to mechanical stimulation in the whisker pad was significantly attenuated in the IAN-X rats (Figure 2G). On the other hand, administration of saline (i.p. or m.i. into the motV) in the IAN-X rats did not affect the head-withdrawal threshold (Figure 2G). In addition, there was no significant influence of MC or saline on the head-withdrawal threshold on the side contralateral to the IAN-X or sham operation (Figure 2H).

### Modulation of masticatory jaw movements

**Figure 3:**
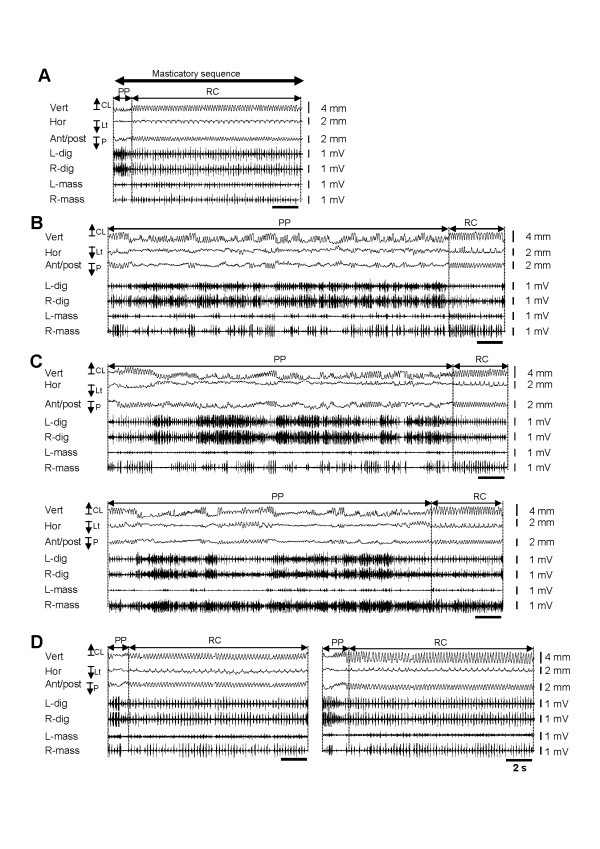
**Typical examples of masticatory sequences.** A entire masticatory sequence in sham rats **(A)**, IAN-X rats **(B)**, IAN-X + saline rats (i.p.) (**C**, upper graph), IAN-X + saline (m.i.) (**C**, lower graph), IAN-X + MC (i.p.) rats (**D**, left sided graph) and IAN-X + MC (m.i.) rats (**D**, right sided graph). The recordings include horizontal, anterior/posterior and vertical components of the jaw-movement trajectories and EMG activities of the digastric and masseter muscles. Whole masticatory sequences were defined as the period of intake of food to just before swallowing and was further divided into two masticatory periods (i.e., the preparatory period and the rhythmic-chewing period) based on the jaw-movement trajectories and the EMG activities of jaw muscles. It is notable that the masticatory movement was modulated after IAN-X, and administration of MC in IAN-X rats significantly improved the masticatory behavior.

During masticatory jaw movements recording in sham rats, rats were stayed in the plastic cage without any sign of discomfort. Each animal could readily ingest the food when the food was presented in front of the rat’s mouth. As described in the Methods section, the entire masticatory sequence was divided into the PP and RC periods on the basis of EMG activities and jaw-movement patterns (Figure 3A). The PP period was characterized by the several cycles of simple open-close movements with irregular cycle duration following the initial jaw opening. During the RC period, the animal began to crush and grind the food between the upper and lower molars when the food was transferred to the posterior part of the mouth. This period needed a longer duration to grind the food compared to food preparation. Unlike from the PP period, the RC period is characterized by the prominent horizontal excursion of the jaw (Figure 3A).

**Figure 4:**
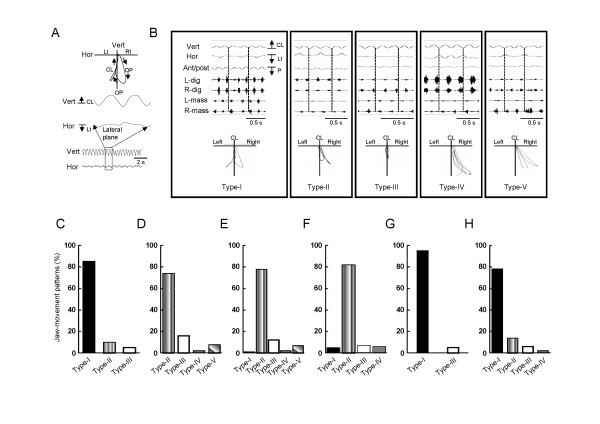
**Masticatory jaw-movement trajectories in lateral plane during the RC period. A**: Jaw-movement trajectories were constructed based on the vertical and horizontal movements. **B**: The jaw-movement trajectories were divided into 4 patterns based on the direction of the horizontal excursion of the jaw, i.e., Type-I (bilateral shift of the jaw), Type-II (jaw moved either contralateral to IAN-X or parallel to the upper jaw), Type-III (no lateral shift of the jaw), Type-IV (jaw moved only contralateral to IAN-X) and Type-V. The jaw movement patterns in the sham rats (**C**), IAN-X (**D**), IAN-X + saline (i.p.) rats (**E**), IAN-X + saline (m.i.) rats (**F**), IAN-X + MC (i.p.) rats (**G**) and IAN-X + MC (m.i.) rats (**H**). In IAN-X rats, bilateral excursion of the jaw (Type-I) was not apparent. The jaw-movement patterns were expressed as the percentage of the total chewing cycle.

**Figure 5:**
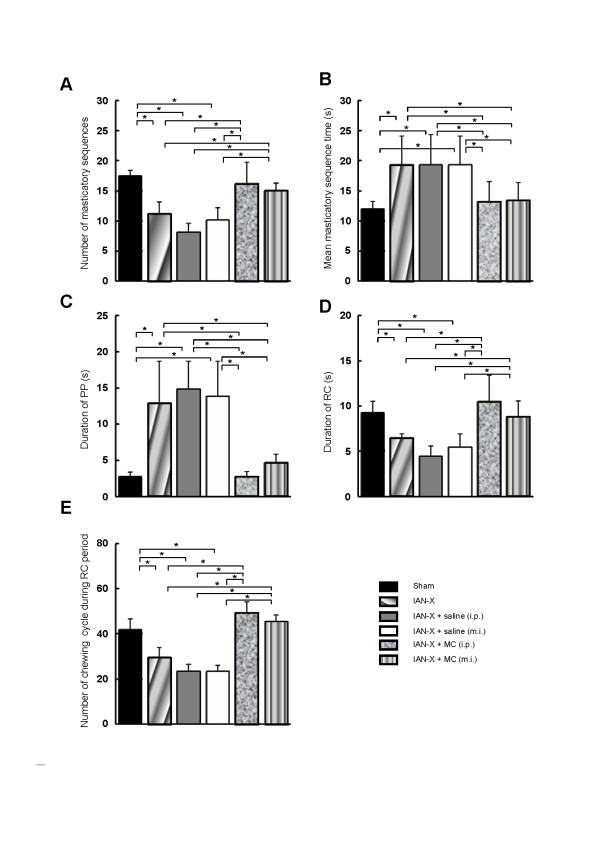
**Effects of IAN-X, IAN-X + MC (i.p. and m.i.) and IAN-X + saline (i.p. and m.i.) administration on the number of the masticatory sequences during 5 min continuous recording (A), mean masticatory sequence time (B), food PP period duration (C) and RC period duration (D), number of the chewing cycle within the RC period (E).** The IAN-X had large effects on the properties of the masticatory behaviors: decrease in the total number of masticatory sequences during 5 min continuous recording, elongation of the total masticatory sequence time as well as the food PP period and shortening the RC period. It is notable that administration of MC improved the masticatory movements in IAN-X rats. (*) denotes the significant difference among the condition of the rats (*p* < 0.05, one ways ANOVA with tukey test).

The masseter muscles were activated in the jaw-closing phase throughout the RC period and the digastric muscles showed prominent rhythmic EMG bursts in relation to the jaw-opening phase (Figure 3A). Rhythmic bilateral shifts of the jaw (Type-I: both right and left side, 3400 (85%) chewing cycle out of 3981) were observed during the RC period in the sham rats (Figure 4B-C). A unilateral shift of the jaw (Type-II: 10%, Type-III: 5%) during this period was rarely observed. During chewing cycles, the animals also performed a series of stable jaw-opening and -closing movements during the RC period, and 42.0 +/− 5.0 chewing cycles were found in each RC period of the masticatory sequence (Figure 5E).

Jaw-muscle activities and jaw-movement patterns in three dimensions of the masticatory sequence were also studied in IAN-X rats (Figure 3B). It was frequently observed that IAN-X rats failed to ingest the food and dropped it. In addition, masticatory jaw movements with irregular large horizontal shifts with prominent jaw-muscle EMG activities were frequently observed during the PP period (Figure 3B and see Table 1). Furthermore, cyclic jaw movements with irregular horizontal excursions of the jaw could be observed during the RC period, indicating that the rats showed considerable difficulty in moving their jaw to the IAN-X side during the RC period (Figures 3B, 4D). The IAN-X rats moved their jaw to either the contralateral side to the IAN-X site (Type-II) or made no horizontal excursion of the jaw (Type-III) during this period (Figure 4B, D). A total of 1689 (74%) chewing cycle out of 2275 (n = 7) was belongs to the Type-II jaw-movement pattern which was different from those in the sham rats (Figure 4C-D). It was also notable that bilateral excursions of the jaw were not apparent in IAN-X rats (Figure 4D). Nonetheless, IAN-X rats seemed to be able to complete the masticatory trails as long as they successfully received and transported the foods to the occlusal surface of the molar teeth. The total number of complete masticatory sequences was significantly shorter in IAN-X rats (11.0 +/− 3.0) compared to sham rats (17.0 +/− 1.0) during 5 min continuous recording (Figure 5A).

**Table 1:** A) Duration of the jaw muscle EMG activities, B) Area of the jaw muscle EMG activities

A)								
Masticatory periods								
	PP				RC			
	Duration of jaw muscles (s)				Duration of jaw muscles (s)			
	Jaw-closing muscles		Jaw-opening muscles		Jaw-closing muscles		Jaw-opening muscles	
Group	R-mass	L-mass	R-dig	L-dig	R-mass	L-mass	R-dig	L-dig
sham (n = 7)	0.06+/-0.01	0.06+/-0.01	0.11+/-0.02	0.11+/-0.02	0.08+/-0.01	0.08+/-0.01	0.10+/-0.02	0.10+/-0.01
IAN-X (n = 7)	0.08+/-0.01	0.08+/-0.01	0.10+/-0.01	0.10+/-0.01	0.09+/-0.01	0.08+/-0.01	0.09+/-0.01	0.09+/-0.01
IAN-X + saline (i.p., n = 5)	0.08+/-0.01	0.07+/-0.01	0.10+/-0.01	0.10+/-0.01	0.09+/-0.01	0.07+/-0.01	0.09+/-0.01	0.09+/-0.01
IAN-X + saline (m.i, n = 5)	0.08+/-0.02	0.07+/-0.01	0.11+/-0.02	0.10+/-0.02	0.09+/-0.01	0.08+/-0.01	0.09+/-0.01	0.08+/-0.02
IAN-X + MC (i.p., n = 7)	0.06+/-0.02	0.07+/-0.01	0.10+/-0.02	0.10+/-0.02	0.07+/-0.01	0.08+/-0.01	0.09+/-0.02	0.09+/-0.01
IAN-X + MC (m.i., n = 5)	0.08+/-0.02	0.07+/-0.02	0.10+/-0.02	0.10+/-0.02	0.10+/-0.01	0.08+/-0.01	0.09+/-0.01	0.09+/-0.01
B)								
Masticatory periods								
	PP				RC			
	Area of jaw muscles (s)				Area of jaw muscles (s)			
	Jaw-closing muscles		Jaw-opening muscles		Jaw-closing muscles		Jaw-opening muscles	
Group	R-mass	L-mass	R-dig	L-dig	R-mass	L-mass	R-dig	L-dig
sham (n = 7)	0.006+/-0.001	0.004+/-0.002	0.008+/-0.003	0.010+/-0.004	0.007+/-0.004	0.006+/-0.002	0.006+/-0.003	0.007+/-0.003
IAN-X (n = 7)	0.008+/-0.006	0.002+/-0.001	0.010+/-0.005	0.013+/-0.005	0.010+/-0.004	0.002+/-0.001^a^	0.010+/-0.005	0.010+/-0.004
IAN-X + saline (i.p., n = 5)	0.008+/-0.006	0.002+/-0.001	0.010+/-0.004	0.010+/-0.005	0.010+/-0.001	0.002+/-0.001^b^	0.010+/-0.005	0.010+/-0.003
IAN-X + saline (m.i, n = 5)	0.009+/-0.003	0.002+/-0.002	0.009+/-0.003	0.011+/-0.005	0.010+/-0.002	0.002+/-0.002^b^	0.011+/-0.003	0.010+/-0.002
IAN-X + MC (i.p., n = 7)	0.010+/-0.006	0.003+/-0.002	0.011+/-0.005	0.012+/-0.005	0.012+/-0.006	0.004+/-0.003	0.012+/-0.004	0.011+/-0.003
IAN-X + MC (m.i., n = 5)	0.006+/-0.003	0.003+/-0.003	0.011+/-0.005	0.012+/-0.004	0.007+/-0.002	0.004+/-0.004	0.010+/-0.003	0.008+/-0.003

The mean masticatory sequence time (19.3 +/− 7.0 s) was significantly elongated in IAN-X rats compared to sham rats (12.0 +/− 1.3 s) (Figure 5B). In each masticatory period, duration of PP period was also significantly longer (12.8 +/− 7.0 s) in the IAN-X rats compared with sham rats (2.75 +/− 1.0 s) (Figure 5C). Furthermore, the duration of RC period (6.4 +/− 0.5 s) was significantly shorter in IAN-X rats than that in sham rats (9.2 +/− 1.3 s) (Figure 5D). The total number of the chewing cycles within the RC period (11.0 +/− 2.0) of a masticatory sequence was also significantly decreased compared to that in the sham rats (17.5 +/− 1.0) (Figure 5E). However, the pattern of the chewing cycle as well as the duration of chewing cycle was not affected by IAN-X during both the PP and RC periods. Also, no significant change in the duration of the jaw-closing and -opening phases was observed during both of these periods (data not shown). In the case of EMG activities, although mean area of the left masseter EMG was significantly smaller in the side ipsilateral to the IAN-X than sham rats, the duration of EMG burst was not significantly different among experimental groups (see Table 1).

It is notable that similar modulatory effect on the masticatory movement was observed at day 3 and 7 after IAN-X (Additional File 2: Figure S1).

To investigate whether microglial cells are involved in the modulation of these oro-facial motor functions, we investigated features of the masticatory jaw movements after MC (both i.p. and m.i. into motV) or saline administration (i.p. and m.i. into motV) in the IAN-X rats (Figure 3C-D). The MC administration (i.p. or m.i.) improved masticatory jaw movements (Figure 3D), but administration of saline (i.p. or m.i.) was ineffective to significantly influence the masticatory movements in IAN-X rats (Figure 3C). Masticatory efficacy increased in IAN-X rats after MC administration, and IAN-X rats could take and ingest the food without any difficulty during the recording session (Figure 3D). In addition, a significant improvement of the jaw-movement patterns was observed after MC administration (IAN-X + MC rats) and approximated the patterns seen in the sham rats (Figure 4B, C, G-H), including rhythmic bilateral excursion of the jaw (Type-I) during the RC period, whereas no significant change in jaw-movement patterns was observed after administration of saline in IAN-X rats (Figure 4E-F).

Figure 5A shows the total number of masticatory sequences during 5 min continuous chewing dramatically increased in MC-injected rats compared to IAN-X rats or IAN-X + saline rats during 5 min continuous recording. In addition, the total masticatory sequence time as well as the duration of the PP and RC periods in IAN-X + MC rats was not significantly different from that in sham rats, but different from those in IAN-X or IAN-X + saline rats (Figure 5B-D). Furthermore, the number of chewing cycle during the RC period of each masticatory sequence was also significantly increased after daily i.p. or m.i. injection of MC in IAN-X rats compared to IAN-X or IAN-X + saline rats (Figure 5E).

## Discussion

This study is the first documentation that trigeminal nerve injury induces nociceptive behaviors associated with alternations of several masticatory patterns, and that microglial cell activation in the prV and motV is involved in modulation of masticatory jaw movements after trigeminal nerve injury, and the present findings are summarized as follows: 1) IAN-X was associated with nociceptive behavior and alternations in masticatory motor functions; 2) Significant increase in the number of activated microglial cells could be observed in the prV as well as in the motV after IAN-X; 3) Changes in masticatory EMG activities and jaw movements in IAN-X rats were attenuated when the microglial cells inhibitor MC was microinjected into the motV or even systemically, and MC also significantly reduced the number of activated microglial cells in IAN-X rats.

### Technical considerations

Using awake freely moving rats allowed us to analyze the modulatory effect of IAN-X during natural mastication. We were therefore able to study in IAN-X rats, the modulation of masticatory jaw movements during both the PP and RC periods. It is very important to analyze natural masticatory jaw movements in freely moving rats in order to clarify mechanisms underlying masticatory dysfunction in IAN-X rats without using drugs (e.g., anesthetics) that could affect CNS neuronal activity.

In addition, the IAN was chosen as the target nerve to prepare the IAN-X model in this study since it is mostly a pure sensory nerve. Therefore, IAN-X model gave us the opportunity to study the effects and underlying mechanisms of a pure sensory nerve lesion without the motor nerve damage on the masticatory function [13,35]. Furthermore, we have previously shown that IAN-X proceeds nociceptive behavior (e.g., lowering the head withdrawal threshold) and trigeminal central sensitization [12-15], and that glial cells are involved in this model of trigeminal neuropathic pain [14,16-19]. Such studies supported the use of this model to study glial cells involvement in masticatory content changes following trigeminal nerve injury.

### Identification of regenerated IAN

It has been reported by the various researchers that the injured primary afferent nerve fibers regenerate at 2–3 weeks [36-38]. In the present study, we have observed a large number of FG-labeled neurons in TG at day 14 after IAN-X but not at day 3 after IAN-X, indicating that the FG labeled TG neurons are the result of FG transportation from the mental skin to the TG. This also indicates that transected IAN was undergoing regeneration at day 14 after IAN-X but not at day 3 after IAN-X [15,35].

### Glial involvement in nocifensive behavior

Our hypothesis was that microglia is involved in developing of neuropathic pain in the whisker pad skin innervated by the 2^nd^ branch of the trigeminal nerve on day 3 after IAN-X, and the neuropathic pain developed in the whisker pad skin is persist at least 14 days after IAN-X, and microglial activation is also involved in developing neuropathic pain in the mental skin on day 14 after IAN-X. Thus, we investigated the head-withdrawal threshold to mechanical stimulation of the whisker pad skin to make sure that the microglial activity is directly related to the development of the neuropathic pain. We have found that mechanical escape threshold was significantly lower than the control on day 3 after IAN-X and lasted more than 14 days after IAN-X. Administration of MC decreased the mechanical hypersensitivity in the whisker pad skin in IAN-X rats. In addition, our immunohistochemical analysis showed that microglial cells were activated on day 3 and lasted at least 14 days after IAN-X. Together with the immunohistochemical data, the present findings indicate that microglia is involved in the development and maintenance of neuropathic pain in the whisker pad skin and mental skin following IAN-X.

### Modulation of masticatory jaw movements

We observed a significant reduction of the head-withdrawal threshold at 14 day after IAN-X, and masticatory jaw movements were strongly modulated at this time period in the freely moving rats (Figure 3B). IAN-X rats showed a longer duration to complete mastication of the food than sham rats, and a considerable difficulty in preparation and transportation of the food (Figures 3B, 5B-C). Although IAN-X rats could not move the jaw ipsilateral to the IAN-X side (Figure 4D), there were no significant changes in the properties of the chewing cycles.

Many researchers have reported that the fundamental pattern of mastication is generated mainly by the central pattern generator (CPG) [2,3,39-42], and the essential core of the CPG lies between the rostral poles of motV and facial motor nuclei [3,40], the region that contains the prV [41,42]. The neurons within the prV receive sensory inputs from muscle spindles, periodontal and other intra-oral mechanoreceptors during masticatory jaw movements, and provide the feedback that is necessary for the modulation of the motor pattern [41,42]. It has been reported that non-noxious inputs from intraoral receptors have no or less modulatory effect on the masticatory jaw movements [43-45], but noxious inputs from intraoral receptors could modulate the masticatory jaw movements [5-11]. The clinical pain or experimental pain from the jaw muscle is associated with the reduction of the agonist muscle activity during mastication [5-7,9,10,46], and an increase in antagonist muscle activity [5,47,48]. For example, noxious inputs after bilateral injection of hypertonic saline into the masseter muscles reduced masseter muscle activities in the agonist phase during mastication [6]. However, there are also several data that are not consistent with above observation. For example, it was shown by some studies that pain did not result in significant reduction in amplitude of the jaw movement during mastication or jaw-closing force or masseter EMG [8,47,49]. In addition, simultaneous increases in the EMG activities of jaw-closing and -opening muscles were observed following the injections mustard oil or glutamate in the temporomandibular joint [11,50,51] or jaw muscles [52]. Furthermore, we observed in the present study that neuropathic pain induced by the regeneration and reinnervation of the IAN-X at 14 day affected the ability to carry out mastication although it did not prevent mastication from occurring (Figure 3B). Such masticatory impairment is characterized by a significant elongation of the total masticatory time, including PP period (Figure 5B, C), but there were no or less changes in the EMG activities both in jaw-closing and -opening muscles (see Table 1). The Possible reasons for the differences between these studies and our study are the functional complexity of the sensorimotor system and the multidimensional nature of pain in the orofacial regions [9].

### Microglial involvement in modulation of masticatory jaw movements

In addition to the importance of the neuronal mechanisms in the initiation and maintenance of the oro-facial pain and in orofacial motor control, there is a considerable evidence that non-neuronal (glial) cells play an important role both in peripheral and CNS neuronal mechanisms underlying pathological pain. Although most of the recent studies have mainly focused on the role of the hyperactive microglial or astroglial cells in pain mechanisms, no data have been reported whether hyperactive glial cells after peripheral nerve injury have any role in modulation of oral motor functions. In view of this and our findings of trigeminal motor neuronal changes and glial cell involvement in IAN-X model of trigeminal neuropathic pain, the present study focused on the possible sites of hyperactive glial cells especially in prV and motV in the modulation of masticatory jaw movements following IAN-X.

Various studies have reported that microglial cells display remarkable changes in their morphology and physiology in response to various stimuli. The fact that microglial cells are hyperactive in the Vc (medullary dorsal horn) and the spinal dorsal horn (see review [14,16-18,53-59] after spinal or trigeminal nerve injury. In addition, some recent studies have shown that microglial cells are activated in the areas surrounding the prV [19] and facial motor nuclei [20] following trigeminal nerve injury. We have also found that the microglial cells are activated both in the prV and motV along with Vc after IAN-X.

Glial cells are known to be hyperactive at different times courses after the nerve injury [14,18,57] such that microglial cells in the Vc are activated around 3 days after the injury and slightly reduced around 14 days [17-19,59]. Consistent with the previous findings, we observed that many Iba1-IR cells are expressed both in the prV and motV and changed in their morphology around 3 days after IAN-X, and the number of hyperactive microglial cells was slightly reduced around 14 days after IAN-X Figure 1A, C, D).

Regarding the role of these hyperactive microglial cells, many researchers have reported that hyperactive microglial cells have functional interactions with neurons in many CNS regions [57,60,61]. Following nerve injury, microglial cells produce various substances (such as TNF-α, IL-1β, IL-6, IL-18, NO and BDNF) that affect the neuronal excitability [58,59]. Although microglial glial cells have been recognized for their role in initiating neuropathic pain, but recent evidence suggests that microglial cells may also play an important role in controlling the activity of the neuronal networks underlying motor function. Regarding this, Mika et al. have shown that blocking of the microglial cell activity significantly improves the recovery of motor behavior (walking patterns) after the chronic constriction injury of the sciatic nerve [28]. In consistent with the above observation, we have found that the efficiency of the masticatory jaw movements is decreased when the microglia cells were hyperactive in the prV and motV. Thus, it is possible that hyperactive microglial cells in the prV and motV release proinlfamatory substances and change in the neuronal excitability of trigeminal motor neurons, and thereby modulates the masticatory jaw movement following IAN-X. Furthermore, we cannot rule out the possibility that microglial cells in the Vc may also participate in modulation of masticatory jaw movements.

### The effect of MC on the masticatory jaw movements

To confirm the possible contribution of the hyperactive microglial cells in the modulation of masticatory jaw movements after IAN-X, we studied the effect of MC on neuropathic pain and motor behaviors recovery. MC, a semisynthetic second generation tetracycline derivative antibiotic with adequate penetration into the brain and cerebrospinal fluid [62-64], has emerged as a potent inhibitor of microglial activation and proliferation. Recently, it has been shown that MC attenuates the mechanical allodynia and central sensitization in several animal models of tissue inflammation [16,17,65], traumatic nerve injury and spinal cord injury [24-33] through the inhibition of the microglial cell activation, and improve the motor functions [28,66]. In consistent with the recent findings, we have found that the injection of minocycline 16 h before the transection and then daily after the transection for 14 days strongly attenuates the development of the neuropathic pain in IAN-X rats. These findings is supported by our immunohistochemical data showing that systemic administration of MC strongly attenuated the microglial cell activation both in the prV and motV on day 14 after IAN-X as well as overcoming the impaired masticatory motor patterns that was typical in this model.

## Conclusions

Our results suggest that the neuropathic pain associated with the regeneration of IAN-X strongly affects mastication. The findings also indicate the possible contribution of hyperactive microglia in the modulation of masticatory motor function after regeneration of the transected IAN and that activation of microglia in the prV and motV may play an important role in modulation, since the microglial inhibitor MC was effective to reduce the severity of neuralgia and the associated nociceptive behaviors and impaired masticatory behaviors. Our data also suggest that MC may be a useful drug to treat the orofacial motor deficits after peripheral nerve injury. It is likely that this study will open new insights for clinicians to diagnose and manage masticatory dysfunction after trigeminal nerve injury.

## Methods

The experiments were carried out in accordance with the guidelines of National Institute of Health Guide for the Care and Use of Laboratory (NIH Publication no. 80–23) revised 1996 and the International Association for the Study of Pain in conscious Animals [67], and were approved by the intramural Animal Care and Veterinary Science Committee of Niigata University. A total of 101 male Sprague–Dawley rats weighing 200–250 g were used in this study. Rats were divided into four groups according to their preparation (sham rats; n = 17, IAN-X rats; n = 42, IAN-X + MC rats; n = 22, IAN-X + saline rats; n = 20).

### Inferior alveolar nerve transection (IAN-X)

Before IAN-X, the site of the transection was marked to avoid the damage of the motor nerve. It has been reported that the IAN entered the mandibular foramen as a large single trunk and runs along the medial aspect of the mandible through the mandibular canal (Additional File 1: Figure S1Aa, Ab) [68]. Before enter to the mandibular foramen, the IAN gives off a motor nerve branch namely the mylohyoid nerve which coursed anteriorly along the lingual surface of the mandible in the mylohyoid groove (Additional File 1: Figure S1Aa, Ab) [69]. Thus, it was decided to expose the IAN by making a small hole on the external surface of the mandibular canal since the IAN within the mandibular canal is almost sensory in nature, and also the injury of this nerve does not affect the peripheral representation of the auriculotemporal, mylohyoid, lingual and maxillary nerves [70].

The methods have been previously detailed [13,35] and only a brief description follows. Rats were initially anesthetized with sodium pentobarbital (initial dose, 50 mg/kg, i.p.). The adequacy of anesthesia was checked repeatedly throughout the surgery by noxious pressing the hind paw to determine if a withdrawal reflex was evoked, and if so, a supplementary dose of sodium pentobarbital was given.

For IAN-X, a small horizontal incision was made on the surface of the facial skin over the masseter muscle and the surface of the alveolar bone was exposed by dissecting the overlying tissues (Additional File 1: Figure S1Ac). The alveolar bone over the IAN was removed slowly with a small round bar (no: 6) and the IAN was exposed (Additional File 1: Figure S1Ac). Then the exposed IAN was lifted up from the mandibular canal without any injury by the angulated thin glass rods and transected just above the angle of the mandible (Additional File 1: Figure S1Ad). The transected IAN was placed back in the mandibular canal without any discernable gap between its cut-ends (Additional File 1: Figure S1Ae). It is notable that the mental and incisor branch of the IAN was not discriminated in the present study.

In the case of sham rats, similar horizontal incision was made on the facial skin, the exposed alveolar bone covering the IAN was removed, and the IAN was exposed and left intact.

### Drug administration

**Figure 6:**
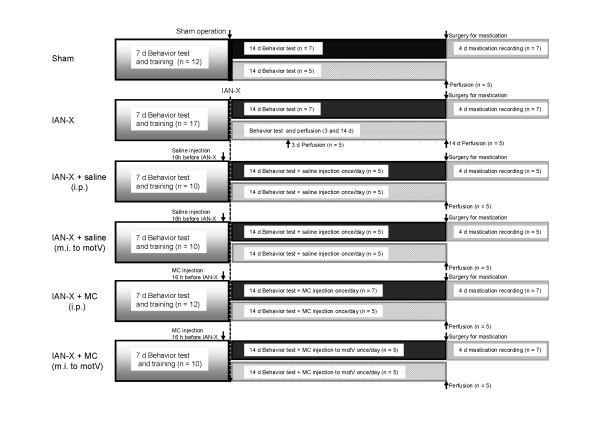
**The time course of the present experiment.** The rats were trained for behavioral testing at least 7 days before any surgical operation and drug or chemical injection. After the training, the rats were prepared for the sham operation or IAN-X, and the behavioral test was conducted for 14 days after the sham operation or IAN-X. In MC (both i.p. and m.i.) or saline (both i.p. and m.i.) injected rats, the drug was injected 16 h before transection, and was administrated once in a daily for 14 days after IAN-X. After behavioral test, the rats were prepared either for a second surgery prior to recording masticatory jaw movement or for perfusion prior to immunohistochemistry. In the case of IAN-X rats, the perfusion was conducted at 3 and 14 day after IAN-X. Note that the masticatory jaw movement was recorded for at least 4 days.

Minocycline (MC) was suspended in isotonic saline. The MC solution (50 mg/kg body weight, Sigma, USA) or vehicle (saline, 1 ml/day) was administrated intraperitoneally (i.p.) [28,34] 16 h before IAN-X, and the injection was then given daily for next 14 days (Figure 6). The influence of MC on the head-withdrawal threshold to mechanical stimulation of the mental skin was tested once daily for 14 days after IAN-X.

In addition, MC or saline was given by microinjection (m.i.) into the motV nucleus (Figure 6). A vertical incision was made over the head skin to prepare a small hole in the left side of over the occipital bone (9.5 mm posterior and 2 mm lateral from the bregma point) for the MC administration into the motV. The needle was inserted (8.2 mm in depth) stereotaxically to the motV through the hole, and the needle head was fixed with adhesive dental acrylic. Then 0.1-0.05 μl of MC was administrated preemptively into the motV 16 h before IAN-X and then daily for the next 14 days (Figure 6).

### Behavioral testing

Rats were trained daily to stay in a plastic cage without any discomfort for 20 min, and protruded their perioral region through a hole made in the wall of the plastic cage as previously described [13,35]. The mechanical head-withdrawal threshold was measured by using a series of von-Frey filaments applied to the mental skin (supplied by the IAN) and the whisker pad skin (supplied by the infra-orbital nerve). One week after the training, rats were capable of receiving mechanical stimulation of the mental skin and whisker pad skin with their perioral regions protruding the nose through the hole for 20 min. Before the start of the training, water was restricted to 50 ml/day for 2 days. In daily sessions, water was used as a reward to train rats to stay in the plastic cage and to drink water through the hole during noxious stimulation of the mental skin and whisker pad skin. The criterion performance was when the rats could keep drinking water for 20 min without escape from noxious stimulation applied by the von-Frey filaments to the mental skin and whisker pad skin. The mechanical head-withdrawal threshold was measured three times both in ascending and descending orders, and the average value was used for analysis. The maximum stimulus intensity differed between rats before training (15–60 g) since each rat had a different head-withdrawal threshold to mechanical stimulation. When a 70 g stimulus was applied to the mental skin or whisker pad skin, all rats showed escape behavior, and so 70 g was the strongest stimulus intensity used for the nocifensive behavioral testing. Such training sessions were continued until the criterion performance was reached (Figure 6). After cession of the training, rats were prepared for sham operation or IAN-X, IAN-X + MC injection, or IAN-X + saline injection. After the surgery, the head-withdrawal threshold to mechanical stimulation was measured once daily for 14 days (Figure 6). In the case of IAN-X rats, when the head-withdrawal threshold was between 2 to 8 g, these rats were prepared for recordings of jaw movements and EMG activities from masseter and digastric muscles (Figure 6).

### Jaw movements and EMG recordings

A total of 46 rats of the 101 rats were chosen at random for the recordings of jaw movements and EMG activities (sham rats; n = 7, 3 day IAN-X rats; n = 5, 7 day IAN-X rats; n = 5, 14 day IAN-X rats; n = 7, 14 day IAN-X + MC (i.p.) rats; n = 7, 14 day IAN-X + MC (m.i.) rats; n = 5, 14 day IAN-X + saline (i.p.) rats; n = 5, 14 day IAN-X + saline (m.i.) rats; n = 5) (Figure 6). Before surgery, the animals were anesthetized with sodium pentobarbital (initial dose, 50 mg/kg, i.p.) and a small amount of lidocaine (2%) was injected into the skin to abolish nociceptive signals by the surgery.

A pair of bipolar multi-stranded, Teflon-coated stainless-steel wire electrodes was implanted bilaterally to record EMG activities from the masseter and anterior digastric muscles. A small cylindrical magnet (4 × 4 mm) was fixed to the animal’s chin with adhesive dental acrylic to record the jaw movements in three dimensions (Vertical, horizontal and anterior/posterior planes). A jaw-tracking system which consisted of four magnetic sensors was attached to the head [71]. The jaw-movement trajectories were traced as movements of the magnet by four magnetic sensors during mastication by the animals. Wires from the electrodes for EMG recordings were led subcutaneously to a connector fixed to the parietal bone.

After the animal had recovered from surgery, the EMG activities of the jaw muscles and jaw movements in three dimensions were recorded during mastication. During the recording sessions, animals ate the test food (pellets) in the plastic cage. Recordings were made daily for 4 days, and each session lasted less than 1 h, and were discontinued if the animals showed discomfort. At the completion of the experiment, the animal was sacrificed by an over-dose of sodium pentobarbital (80 mg/kg, i.p.) administration, and the injection site and EMG electrode locations were confirmed by post-mortem dissection.

EMG activities were amplified using a custom-built AC amplifier (band pass: 0.1–3 kHz), and EMG signals and the jaw movement trajectories were digitized (sampling rate: 2,000/s for EMGs, 300/s for jaw movement) with the Spike2 analysis package (Cambridge Electronic Design Ltd., Cambridge, UK). EMG activities and the jaw-movement trajectories were stored in a hard disc and analyzed offline.

### Data analyses of jaw movements and EMG activities

For the analysis, the following off-line determinations were made. First, the masticatory sequence was defined as the period from the onset of the digastric burst associated with mouth opening to obtain the food (initial jaw opening) to the end of the cyclic jaw movements related to mastication; the duration of this sequence was defined as the masticatory sequence time (Figure 3A). The total number of the masticatory sequences within 5 min of the continuous recording was calculated. Next, each masticatory sequence was divided into two masticatory periods: the preparatory (PP) period (food intake and transportation of food to the occlusal surface) and rhythmic-chewing (RC) period (food is “chewed” by the opposing molars) (Figure 3). The masticatory sequence always started with the food preparation and incision (PP) followed by chewing (RC). The masticatory period was determined on the basis of the jaw-movement patterns (vertical, horizontal and anterior/posterior movements) and the related jaw muscles EMG activity patterns (Figure 3). At the early stage of mastication, irregular cyclic open-close movements with small jaw-closing muscle activities were observed. Since small jaw-closing muscle indicates that strong bit force did not occur, this period is considered as the period of food intake and transportation. The PP period was defined as the period from the onset of the digastric burst related to the initial jaw opening to the start of RC period (Figure 3). Following the PP period, the jaw-movement pattern changed abruptly to that with clear lateral shift of the jaw in the jaw-closing phase (Figure 3). Such jaw movements were characterized by large rhythmic jaw-closing muscle bursts, indicating that the food is “chewed” by the opposing molars, was termed as RC. Thus, this period was defined as the period from the onset of the marked masseter burst along with a prominent lateral shift of the jaw to the end of the cyclic jaw movements related to mastication (Figure 3). The duration of each masticatory period was calculated, and the jaw-movement trajectories in the lateral plane (combination of vertical and horizontal jaw movements) were analyzed during the RC period (Figure 4A). The jaw movement patterns was divided into five types (i.e., Type-I, Type-II, Type-III, Type-IV and Type-V) on the basis of the horizontal excursion of the lower jaw (Figure 4B). For the chewing cycle, the duration from the point of maximum jaw opening to the point of next maximum opening in the vertical component of the jaw-movement trajectories was calculated, and termed chewing cycle duration. Furthermore, the following variables were analyzed for each chewing cycle in each masticatory period: 1) the number of chewing cycles within the RC period, 2) the duration of chewing cycles and the duration of jaw-closing and jaw-opening phases. The jaw-closing phase was defined as that from maximum jaw opening to minimum jaw opening, and the jaw-opening phase as that from the end of the jaw-closing phase to the following maximum jaw opening.

The duration of the masticatory sequence, each masticatory period and chewing cycles were compared among animal groups. In addition, more detailed analysis was performed on EMG activities to evaluate if any changes occurred. Analysis of EMG activities involved assessment of the mean duration and area of the jaw-muscles activity. For this, 10 complete masticatory sequences were randomly selected for the analysis of the EMG for each of the day. First, the averaged EMG value for each parameter was calculated for each of the day. Then, the mean value for 4 days was averaged together, and the mean of the mean value of the EMG was obtained.

### Histology

In the case of the IAN-X + MC (m.i.) and IAN-X + saline (m.i.) rats, 4 M direct blue or pontamine blue was microinjected into the motV after completion of the recordings, to locate the area of the microinjection. Then animals were deeply anesthetized with sodium pentobarbital (80 mg/kg, i.p.) and perfused through the heart with the 0.9% saline (500 ml) and 4% paraformaldehyde (500 ml). The block of the brain was preserved in 4% paraformaldehyde for 3–4 days and then 20% sucrose in 0.01 M phosphate buffered saline (PBS, pH 7.4) for another 3–4 days in 4 C temperature. Then 30 μm frozen sections were cut and stained with cresyl violet to confirm the injection site (Figure 1B).

### Immunohistochemistry

On day 3 or 14 after IAN-X or on day 14 after IAN-X + MC (both i.p. and m.i.) or IAN-X + saline (both i.p. and m.i.), or on day 14 after sham operation, 5 rats were chosen from each group at random for the immunohistochemical study. Rats were anesthetized with sodium pentobarbital (80 mg/kg body weight, i.p.) and transcardially perfused with the 0.9% isotonic saline (500 ml) and sequentially with fresh 4% paraformaldehyde in 0.1 M phosphate buffer (PB, pH 7.4, 500 ml). The medulla and the pons were removed and postfixed in 4% paraformaldehyde for 3 days at 4 °C and then the tissues were transferred to 20% sucrose (w/v) in 0.1 M PBS for several days for cryoprotection. Serial frozen sections (30 μm thickness) were made through the medulla and pons, and collected in 0.01 M cold PBS. Single immunohistochemical procedures were performed at room temperature unless otherwise stated. Free-floating sections were washed with 0.01 M PBS (pH 7.4) for 10 min, and kept by the blocking solutions containing 5% normal goat serum and 0.1% triton X for 2 h at room temperature with agitation. After 2 h, the sections were incubated in rabbit polyclonal anti-Iba1 antibody (for labeling microglia, 1:1000; Wako, Japan) for 2 days at 4 °C with agitation. Two days after that, sections were washed with PBS and incubated in ant-rabbit Alexa Fluor 568 IgG (1:1000; Invitrogen) for 3 h at room temperature with agitation. Then sections were washed in PBS and were mounted and cover-slipped. Images were captured by using the Keyence BZ-8000 fluorescence Microscope (Keyence, Japan). The present study was mainly focused in prV and motV, since previous studies have confirmed the activation of Iba1/microglia in the Vc [17-19]. Thus, the total number of microglia in the prV and motV were counted by using a computer-assisted imaging analysis system (NIH Image, version 1.61).

### Fluorogold (FG) tracing

FG tracing study was conducted on the trigeminal ganglion (TG) in 20 rats (sham: n = 5, 3 day IAN-X: n = 5, 7 day IAN-X: n = 5, 14 day IAN-X: n = 5). Rats were initially anesthetized with the sodium pentobarbital (50 mg/kg, i.p.), and 10 μl of 4% FG was injected subcutaneously in the mental skin 1.5-2.0 mm lateral and 1.0-1.5 mm below the edge of the lower lip and at a depth of 5 mm from the surface. Two days after the FG injection, rats were deeply anesthetized with the same anesthetics and transcardially perfused with the 0.9% isotonic saline (500 ml) and sequentially with fresh 4% paraformaldehyde in 0.1 M PB (500 ml). The TG was removed and postfixed with the 4% paraformaldehyde for 2 days and the tissue was then transferred to 20% sucrose (wt/vol) in 0.01 M PBS for several days for cryoprotection. Serial frozen sections (16 μm thickness) were made with a freezing microtome. The sections were washed in PBS, serially mounted on gelatin-coated slides and cover-slipped. Images were captured by using the Keyence BZ-8000 fluorescence Microscope (Keyence, Japan). The FG-labeled neurons with clear nuclei were counted and their areas were measured in the root of the third branch of the trigeminal nerve by using computer-assisted imaging analysis system (NIH Image, version 1.61).

### Statistical analysis

One way ANOVA with Dunnett’s test was conducted during comparing the mechanical escape threshold between pre and post IAN-X, pre and post IAN-X + MC, or pre and post IAN-X + saline. Other statistical analysis was conducted by using one way ANOVA with Tukey Test. p < 0.05 was considered significant.

## Abbreviations

IAN, inferior alveolar nerve; IAN-X, inferior alveolar nerve transection; prV, trigeminal principal sensory nucleus; motV, trigeminal motor nucleus; Vc, trigeminal subnucleus caudalis; Iba1-IR, Iba1-immunoreactive; MC, minocycline; i.p., intraperitoneal injection; m.i., microinjection; PB, phosphate buffer; PBS, phosphate-buffered saline; FG, fluorogold; TG, trigeminal ganglion; PP, preparatory period; RC, rhythmic-chewing period; EMG, electromyography; CNS, central nervous system; CPG, central pattern generator; Ver, vertical component of the jaw; Hor, horizontal component of the jaw; Ant/post, anterior/posterior component of the jaw; R-dig and L-dig, right and left digastric muscles; R-mass and L-mass, right and left masseter muscles; ANOVA, analysis of variance.

## Competing interest

The authors declare that they have no competing interests.

## Author’s contribution

All authors read and approved the final manuscript. RMM and HMZ carried out the experiments and data analysis, and were equally contributed for conducting experiment. YY, KY and BJS provided data interpretation and helped to finalize the manuscript. JK and KI conceptualized the hypothesis, designed and supervised the experiments, directed data analysis, and finalized the manuscript.

## Supplementary Material

Additional file 1 Figure S1. Click here for file

Additional file 2 Figure S2. Click here for file
