# Supplementary figures and images for: The effect of minocycline on the masticatory movements following the inferior alveolar nerve transection in freely moving rats
Source: Mol Pain. 2012 Apr 20;8:27. doi: 10.1186/1744-8069-8-27 (PMC3416740; doi:10.1186/1744-8069-8-27)

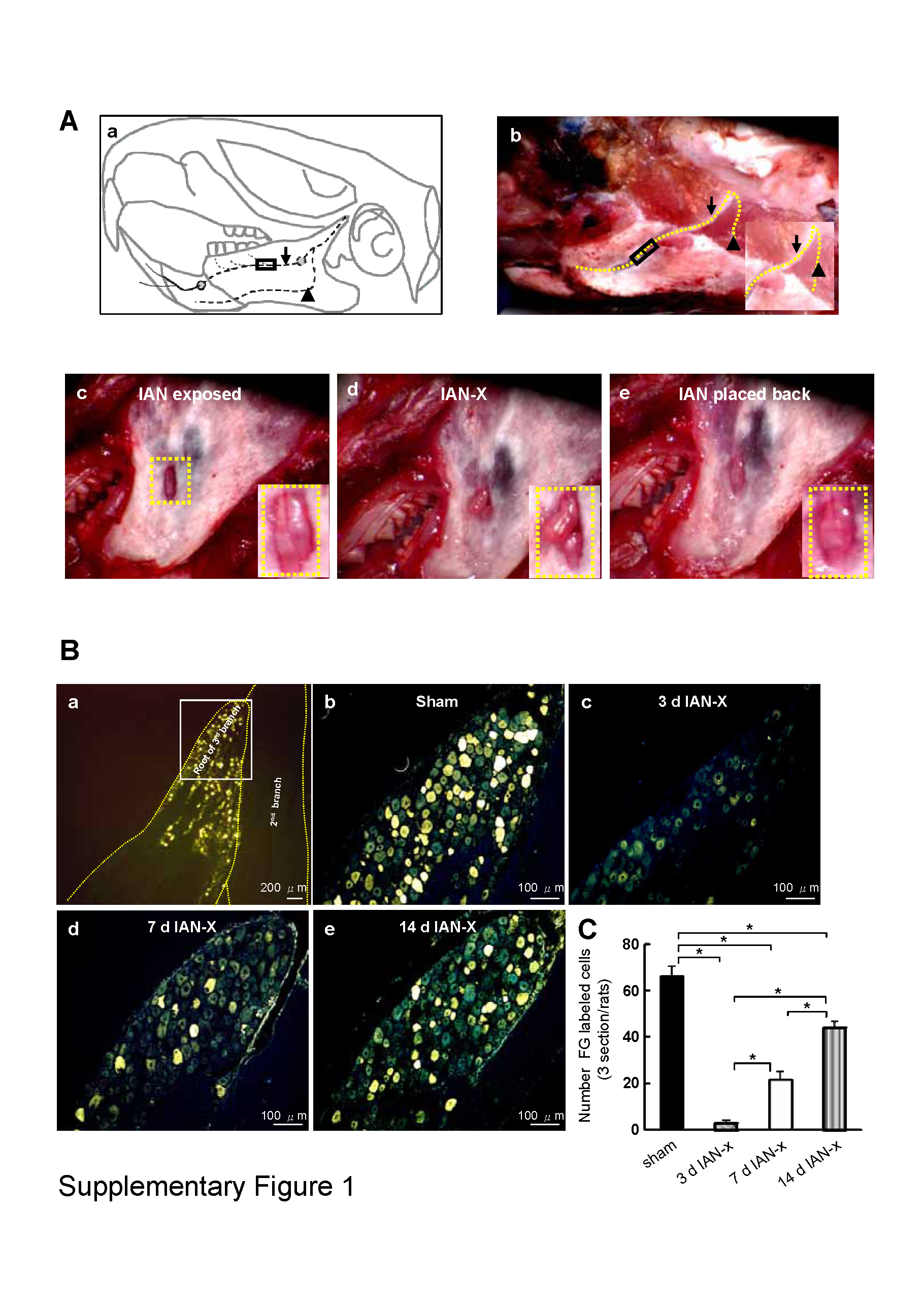

Supplement: Additional file 1 — Figure S1. [file 1744-8069-8-27-S1.tiff]

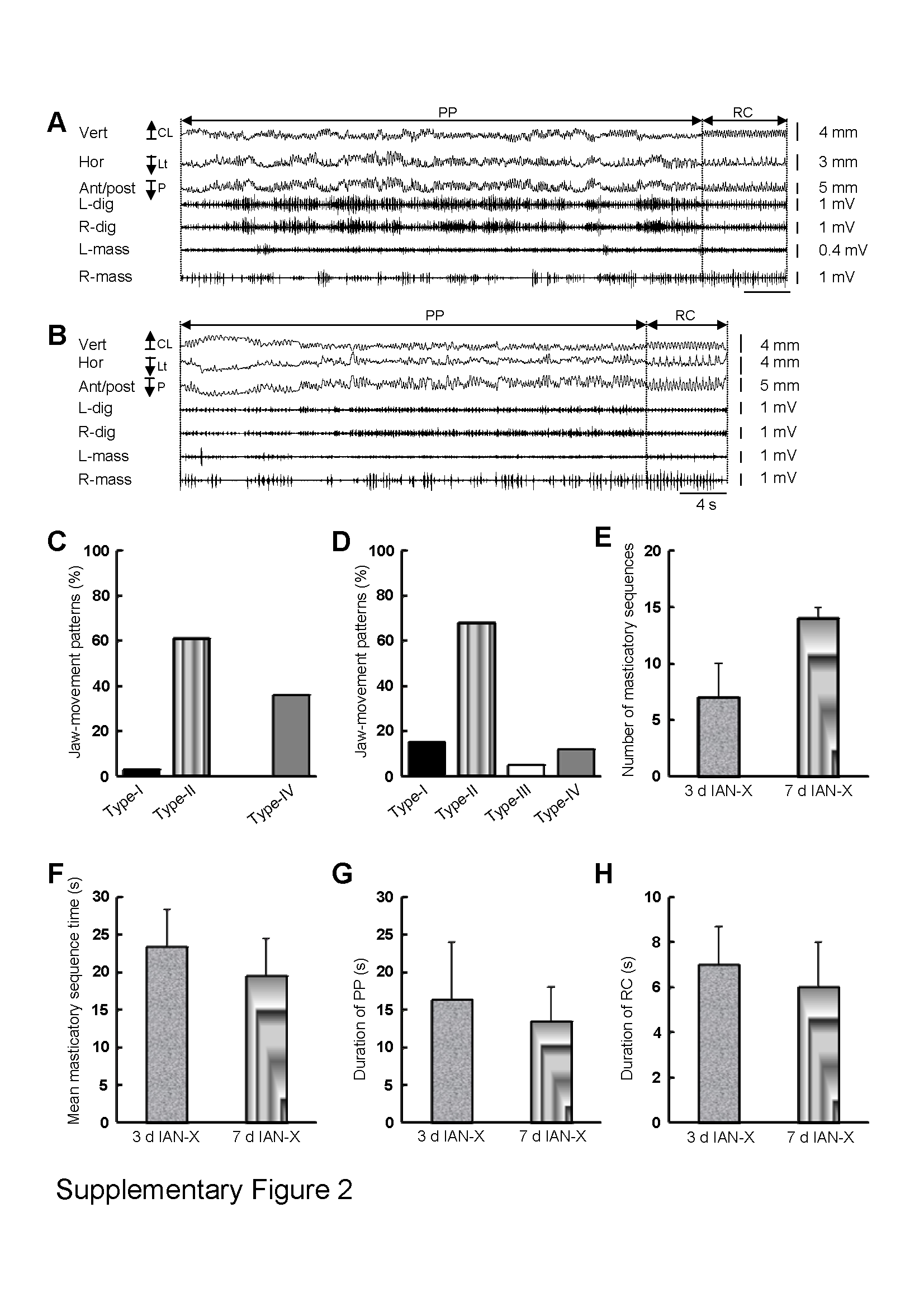

Supplement: Additional file 2 — Figure S2. [file 1744-8069-8-27-S2.tiff]
